# Clinical Factors and Viral Load Influencing Severity of Acute Hepatitis A

**DOI:** 10.1371/journal.pone.0130728

**Published:** 2015-06-19

**Authors:** Hyun Woong Lee, Dong-Yeop Chang, Hong Ju Moon, Hye Young Chang, Eui-Cheol Shin, June Sung Lee, Kyung-Ah Kim, Hyung Joon Kim

**Affiliations:** 1 Department of Internal Medicine, Chung-Ang University College of Medicine, Seoul, Korea; 2 Laboratory of Immunology and Infectious Diseases, Graduate School of Medical Science and Engineering, KAIST, Daejeon, Korea; 3 Department of Internal Medicine, Ilsan Paik Hospital, Inje University College of Medicine, Goyang, Korea; University of Sydney, AUSTRALIA

## Abstract

**Background and Aims:**

Clinical manifestations of hepatitis A virus (HAV) infection vary from mild to fulminant hepatic failure (FHF) in adults. We investigated the relationship between laboratory findings, including viral load, and clinical outcomes in patients with acute hepatitis A (AHA) and evaluated predictive factors for severe acute hepatitis (s-AH).

**Methods:**

We analyzed the clinical manifestations of AHA in 770 patients. Patients with a prothrombin time (PT) of less than 40% of normal were classified as s-AH and included 4 patients with FHF, 11 patients with acute renal failure, and 3 patients with prolonged jaundice (n = 128). Other patients were defined as mild acute hepatitis (m-AH) (n = 642). Serum samples were obtained from 48 patients with acute hepatitis A. Among them, 20 with s-AH, and 28 with m-AH, were tested for HAV RNA titer.

**Results:**

In a multivariate analysis, age (HR = 1.042, *P* = 0.041), peak creatinine (HR = 4.014, *P* = 0.001), bilirubin (HR = 1.153, *P* = 0.003), alanine aminotransferase (ALT) (HR = 1.001, *P*<0.001), initial lactate dehydrogenase (LDH) (HR = 1.000, *P* = 0.045) and total cholesterol (HR = 0.978, *P*<0.001) were independent factors for s-AH. Serum HAV RNA was detected in 20/20 (100%) patients with s-AH and 22/28 (78.6%) patients with m-AH. In a multivariate analysis of the 48 patients who were tested for HAV RNA, peak ALT (HR = 1.001, *P* = 0.004) and HAV RNA titer (HR = 2.076, *P* = 0.012) were independent factors for s-AH.

**Conclusions:**

Clinical factors including age, peak creatinine, bilirubin, ALT, initial LDH and total cholesterol were independent factors for s-AH in a multivariate analysis. In particular, HAV load strongly correlated with the severity of hepatitis A.

## Introduction

Hepatitis A virus (HAV) is a positive-strand RNA virus of the *Picornaviridae* family [1]. It is spread by fecal-oral transmission [2,3]. Although HAV is controlled by vaccination, it is still a public health problem [4]. Improved hygiene has reduced the incidence of hepatitis A infection in developed countries. In Korea, the adult population has not encountered HAV exposure due to improved sanitation according to socioeconomic status. Hence, the age of infection has gradually changed from childhood to adulthood. Since 2005, Korea has experienced several hepatitis A outbreaks in young adults. In children, the disease is usually asymptomatic and mild [5,6]. In contrast, adult clinical manifestations vary from self-limited, anicteric infection to fulminant hepatic failure (FHF) [7,8]. FHF is a rare complication occurring in less than 1% of patients with acute hepatitis A (AHA), as reported previously [9]. A retrospective, observational study in a single referral center reported that the incidence of FHF in Korea increased from 3.2% in 2005 to 13.0% in 2008 [10]. Some reports have shown that the incidence of severe hepatitis is high in patients with underlying chronic liver disease and those older than 40 years [11,12]. However, whether this severe acute hepatitis A (s-AH) is due to a strong immunologic factor or other virologic factors is not clear.

Although reports on viral load during HAV infection are rare, a low or undetectable HAV load has been associated with FHF. This result suggests that FHF is associated with a strong immunological response [9]. In addition, Sainokami et al. reported that the HAV viral load was closely correlated with liver damage and disease severity in mild acute hepatitis (m-AH) but not in severe acute hepatitis (s-AH). These results indicate that other factors, such as old age or alcohol intake, are more important in predicting s-AH and FHF [13]. Despite advances in the virological study of HAV, the disparity between HAV viral load and clinical manifestation has not been completely resolved. The poor understanding of s-AH pathogenesis led us to study its clinical manifestation and predictive factors.

The aims of this study were to investigate the relationship between laboratory findings, including viral load, and clinical outcomes in patients with AHA and to evaluate predictive factors for s-AH.

## Materials and Methods

### Study population

A total of 770 patients diagnosed with AHA who visited Chung-Ang University Hospital and Ilsan Paik Hospital between Feb. 2009 and Sep. 2012 were enrolled in the study. Patients enrolled in the study met the following inclusion criteria: they were 18–65 years of age; they tested positive for serum anti-HAV immunoglobulin (IgM) antibodies and they had elevated serum alanine aminotransferase (ALT) in conjunction with jaundice, fatigue, nausea and vomiting.

### Methods and Definitions

Age, gender, complete blood count, liver function test, and coagulation test were collected from all patients at admission. The following blood tests were performed regularly during the hospital stay. Clinical severity was determined by peak laboratory values.

Patients with a prothrombin time (PT) of less than 40% of normal were classified as “severe acute hepatitis (s-AH)” and included 4 patients with FHF, 11 patients with acute renal failure (ARF), and 3 patients with prolonged jaundice. FHF was defined as the development of hepatic encephalopathy within 4 weeks after disease onset. ARF was defined as elevated serum creatinine levels (>4.0mg/dL) and oliguria (<500mL daily output) in patients with no history of chronic renal disease. Other patients were defined as “mild acute hepatitis (m-AH)” [13,14]. The study protocol conformed to the ethical guidelines of the 1975 Declaration of Helsinki and was approved by the ethics committee of Chung-Ang University, and written informed consent was obtained from each patient [C2008079(182)].

### Serologic markers

All the patients were confirmed positive for IgM anti-HAV and were negative for other markers of acute infection, including hepatitis B surface antigen, IgM anti-HBc, IgM anti-HEV, IgM anti-VCA (Epstein-Barr virus), anti-HCV, anti-HIV, IgM anti-cytomegalovirus (CMV) and IgM anti-herpes simplex virus (HSV).

### HAV RNA titer

Serum samples were obtained from 48 patients within 24 hours after admission and were stored at -8°C until assayed. Samples were collected from 20 patients with s-AH and 28 patients with m-AH. HAV RNA was quantified by using a real-time PCR assay that was based on Taq Man chemistry. Real-time PCR was carried out with the LightCycler Hepatitis A virus quantification kit (Roche Diagnostics, GmbH Mannheim, Germany) according to the manufacturer’s instructions.

### Statistical analysis

All parameters recorded within 24 hours after admission were analyzed. All data were expressed as mean ± standard deviation (SD). We assessed all variables with univariate and multivariate analyses. Fisher exact test was used to compare 2 nominal variables (s-AH and m-AH), and t-test was used for continuous variables (age, alanine aminotransferase, bilirubin, prothrombin time, and HAV RNA titer). Multivariate analyses were performed by using logistic regression analysis to identify factors associated with s-AH. All statistical analyses were performed with Statistical Package for the Social Sciences (SPSS Inc. Chicago, IL, USA), version 12.0.

## Results

### Clinical characteristics and outcomes of patients with hepatitis A

The mean age of the 770 patients was 31 years (range, 18–65 years), and 481 (62%) were men. Aminotransferase activity peaked within 7 days after admission. The mean peak ALT was 3,147 IU/mL (range, 117–10,650 IU/mL). The mean peak bilirubin was 6.0 mg/dL (range, 1.0–39.5 mg/dL). Four patients were diagnosed with FHF (2 males, 33 and 50 years old, and 2 females, 25 and 26 years old), and all had signs of grade 4 encephalopathy at admission. Three of these patients died before a donor liver could be found, and only one female fully recovered after liver transplantation. Acute renal failure (ARF) developed in 11 patients. Among them, 6 patients underwent hemodialysis and completely recovered. None of the patients died of acute renal failure or other complications during the study period. Jaundice was sustained without liver failure for 6 months after admission in 3 patients (all males, ages 29, 31 and 48 years old). The peak bilirubin levels were 39.5, 34.9 and 30.5 mg/dL, respectively. The other 752 patients recovered spontaneously with a marked decrease in alanine aminotransferase (ALT) activity and clotting factor normalization within 6 weeks after admission. Hepatitis A did not recur during follow-up in any of the patients.

A total of 16 patients had comorbid conditions, including 7 with diabetes mellitus, 4 with chronic hepatitis B, 3 with alcoholic liver disease and 2 with non-alcoholic fatty liver disease. None of the patients were co-infected with HCV. They all recovered spontaneously without complications.

### Clinical differences between mild and severe acute hepatitis A

The clinical and laboratory characteristics of the m-AH (n = 642) and s-AH (n = 128) groups are compared in Table 1. The mean age was higher in the s-AH group than in the m-AH group (33 *vs*. 30, *P* = 0.001). The incidence of s-AH was higher in patients over 40 years than in patients under 40 years (35.8% *vs*. 15.2%, *P* < 0.001).

**Table 1:** Clinical characteristics of 770 patients with acute hepatitis A.

Variables	s-AH (N = 128)	m-AH (N = 642)	*P* value
Male:Female (%)	84:44 (65.6:34.4)	397:245 (61.8:38.2)	0.419
Mean age (years)	33 (18–65)	30 (18–54)	0.001
Age group (%)			
≤ 30 years	51 (13.1)	338 (86.9)	
31–40 years	58 (17.7)	270 (82.3)	
41–50 years	15 (32.6)	31 (67.4)	
51–60 years	2 (40.0)	3 (60.0)	
61 years≤	2 (100)	0 (0)	
Peak serum ALT (IU/L)	5,478 (124–10,650)	2,682 (117–9,620)	<0.001
Peak serum bilirubin (mg/dL)	8.3 (2.2–39.5)	5.5 (1.0–18.8)	<0.001
Peak serum creatinine (mg/dL)	1.6 (0.5–12.6)	0.9 (0.4–1.7)	<0.001
Initial CRP(mg/L)	21.6 (2.3–89.0)	16.4 (0.4–115.1)	0.011
Initial LDH (IU/L)	3,149 (174–15,720)	1,101 (125–16,470)	<0.001
Initial total cholesterol (mg/dL)	94 (40–211)	124 (53–377)	<0.001
Initial platelet (10^6^/L)	138 (51–344)	177 (32–516)	<0.001

NOTE. Values are given as mean (range)

Abbreviation: s-AH, severe acute hepatitis A; m-AH, mild acute hepatitis; ALT, alanine aminotransferase; CRP, C-reactive protein; LDH, lactate dehydrogenase.

The peak ALT level and bilirubin levels were significantly higher in the s-AH group than in the m-AH group (5,478 *vs*. 2,682, *P* < 0.001; 8.3 *vs*. 5.5, *P* < 0.002). In addition, peak creatinine, C-reactive protein (CRP), lactate dehydrogenase (LDH), total cholesterol and serum platelet levels differed significantly between the two groups (Table 1).

In a multivariate analysis, age (HR = 1.042, 95% CI: 1.002–1.084, *P* = 0.041), peak creatinine (HR = 4.014, 95% CI: 1.706–9.442, *P* = 0.001), bilirubin (HR = 1.153, 95% CI: 1.050–1.265, *P* = 0.003), ALT (HR = 1.001, 95% CI: 1.000–1.001, *P* < 0.001), initial LDH (HR = 1.000, 95% CI: 1.000–1.000, *P* = 0.045) and total cholesterol (HR = 0.978, 95% CI: 0.967–0.989, *P* < 0.001) were independent factors for s-AH (Table 2). In addition, the peak ALT and initial LDH levels had strongly significant, inverse correlations with peak PT (*r* = -0.699, *P <* 0.001 and *r* = -0.480, *P <* 0.001). The total cholesterol and platelet levels had significant, positive correlations with peak PT (*r* = 0.494, *P <* 0.001, *r* = 0.519, *P <* 0.001). However, peak bilirubin level was weakly correlated with peak PT (*r* = -0.153, *P <* 0.001).

**Table 2:** Multivariate analysis of predictive factors associated with severe acute hepatitis A.

Variables	HR	95% CI	*P* value^*^
Mean age (years)	1.042	1.002–1.084	0.041
Peak serum ALT (IU/L)	1.001	1.000–1.001	<0.001
Peak serum bilirubin (mg/dL)	1.153	1.050–1.265	0.003
Peak serum creatinine (mg/dL)	4.014	1.706–9.442	0.001
Initial CRP(mg/L)	0.984	0.968–1.001	0.061
Initial LDH (IU/L)	1.000	1.000–1.000	0.045
Initial total cholesterol (mg/dL)	0.978	0.967–0.989	<0.001
Initial platelet (10^6^/L)	1.002	0.996–1.008	0.463

NOTE. ^*^P-value from logistic regression models

Abbreviation: HR, hazard ratio; CI, confidence interval; ALT, alanine aminotransferase; CRP, C-reactive protein; LDH, lactate dehydrogenase.

### Hepatitis A viral load in relation to severity of the disease

To further investigate the possible relationship between HAV viral load and other clinical variables, we performed real-time quantitative polymerase chain reaction (PCR) on stored sera from 48 patients. Serum HAV RNA was detected in 20/20 (100%) patients with s-AH and 22/28 (78.6%) patients with m-AH. Mean serum HAV RNA values at admission were 4.7 log_10_ copies/ml in the 20 patients with s-AH, and 3.3 log_10_ copies/ml in the 22 patients with m-AH *(P =* 0.006). Among the 20 patients with s-AH, 2 patients with FHF had high viral titers (4.9 and 5.3 log_10_ copies/ml).

The clinical and laboratory characteristics of the m-AH (n = 28) and s-AH (n = 20) groups are compared in Table 3. Age and gender did not differ significantly between the m-AH and s-AH groups. The peak ALT level and HAV RNA titer were significantly higher in the s-AH group than in the m-AH group (4,296 *vs*. 2,055 IU/L, *P* < 0.001; 4.7 *vs*. 3.3 log_10_ copies/ml, *P* = 0.006). In accordance with previous results, C-reactive protein (CRP), lactate dehydrogenase (LDH), total cholesterol and serum platelet levels differed significantly between the two groups (Table 3).

**Table 3:** Univariate and multivariate analyses for predictive factors associated with severe acute hepatitis A among 48 patients who were tested for HAV RNA titer.

Variables	s-AH	m-AH	Univariate	Multivariate	
	N = 20	N = 28	*P*-value	HR (95% CI)	*P*-value^*^
Male:Female (%)	12:8 (60.0:40.0)	15:13 (53.6:46.4)	0.658		
Mean age, years (range)	32 (26–42)	30 (18–45)	0.584		
Peak serum ALT, IU/L (range)	4,296 (1,339–10,520)	2,055 (255–5,490)	< 0.001	1.001 (1.000–1.001)	0.004
Peak serum bilirubin, mg/dL (range)	5.7(2.2–10.8)	4.8 (1.0–11.7)	0.214		
Peak serum creatinine, mg/dL (range)	0.9 (0.5–1.3)	0.8 (0.5–1.3)	0.318		
Initial CRP, mg/L (range))	24.6 (3.6–89.0)	7.1 (0.9–87.6)	0.022	1.352 (0.853–2.144)	0.199
Initial LDH, IU/L (range)	2,856 (225–15,720)	568 (166–13,510)	0.037	1.001 (0.999–1.003)	0.307
Initial total cholesterol, mg/dL (range)	109 (68–175)	132 (66–234)	0.023	0.936 (0.849–1.031)	0.181
Initial platelet, 10^6^/L (range)	138 (80–338)	202 (111–504)	0.035	0.998 (0.977–1.027)	0.881
HAV RNA, log_10_ copies/mL (range)	4.7 (2.2–7.1)	3.3 (0–5.4)^†^	0.006	2.076 (1.175–3.669)	0.012

NOTE. Values are given as mean (range)

^*^P-value from logistic regression models

^†^HAV RNA was not detected in 6 of 28 patients with m-AH.

Abbreviation: s-AH, severe acute hepatitis A; m-AH, mild acute hepatitis; HR, hazard ratio; CI, confidence interval; ALT, alanine aminotransferase; CRP, C-reactive protein; LDH, lactate dehydrogenase.

**Fig 1:**
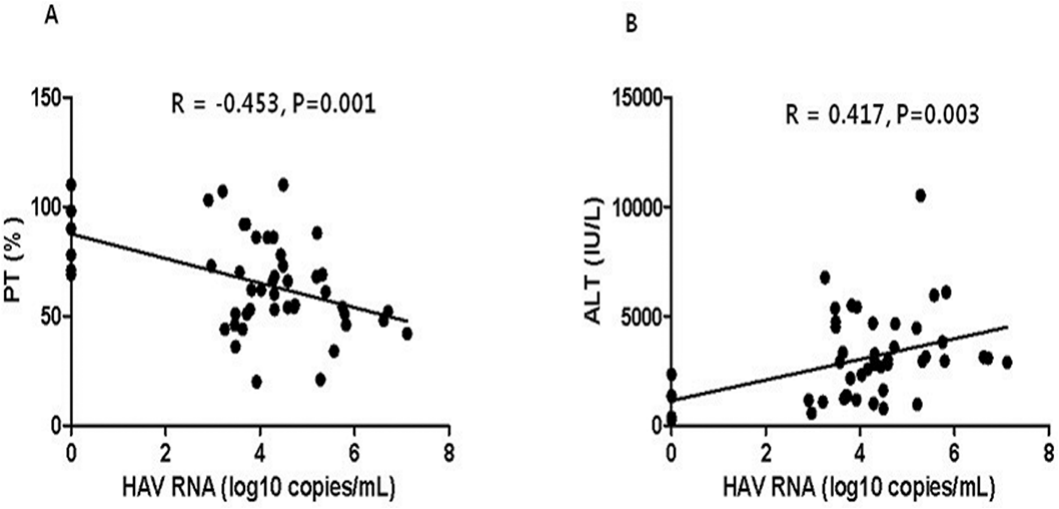
Viral load showed a significant inverse correlation with serum peak PT levels (A, *r* = -0.453, *P* = 0.001) and a significant direct correlation with serum peak ALT (B, *r* = 0.417, *P* = 0.003).

In a multivariate analysis, peak ALT (HR = 1.001, 95% CI: 1.000–1.001, *P* = 0.004) and HAV RNA titer (HR = 2.076, 95% CI: 1.175–3.669, *P* = 0.012) were independent factors for s-AH (Table 3). In addition, viral load showed a significant, inverse correlation with serum peak PT levels (*r* = -0.453, *P* = 0.001) and a significant, direct correlation with serum peak ALT (*r* = 0.417, *P* = 0.003) (Fig 1).

## Discussion

The incidence of hepatitis A infection has been significantly reduced in developed countries by improved hygiene. However, symptomatic AHA has been increasing in developing countries [15–17]. The adult population in these countries has a decreasing rate of exposure because of improved sanitation [18]. In early childhood, hepatitis A infection is usually asymptomatic or mildly symptomatic. In contrast, the spectrum of clinical manifestations in adults varies from mild hepatitis to FHF [4]. The incidence of HAV infection in childhood has decreased, but symptomatic infection has increased in adults. According to a recent report, FHF is the main complication of HAV infection and occurs in 0.91% of patients, and the mortality rate or liver transplantation was 0.47%. The incidence of FHF was slightly lower in our study (4 patients, 0.5%)

Previous studies have suggested that HAV disease severity was related to age [19]. Although old age appeared to be linked to increased AHA severity, it was not an independent factor in multivariate analyses [9]. However, in our study, age was an independent factor for s-AH in the multivariate analysis. Even if the number of patients (n = 53, 6.9%) over 40-years-old was small due to natural immunity against HAV, the rate of s-AH was higher in those patients than in patients under 40 (35.8% *vs*. 15.2%, *P* < 0.001).

In an AHA outbreak area, HAV-associated ARF is not uncommon. Jung et al. reported that patients with HAV-associated ARF had higher ALT levels and prolonged PTs [20]. Our results suggest that peak creatinine is also an important risk factor for s-AH. However, the peak creatinine level was weakly correlated with the peak PT (*r* = -0.121, *P =* 0.001). In addition, the peak bilirubin level was also weakly correlated with the peak PT (*r* = -0.153, *P <* 0.001). These results suggest that serum bilirubin and creatinine levels are not always high in patients with s-AH even though they were independent factors for s-AH.

The peak ALT and initial LDH levels had strongly significant, negative correlations with the peak PT (*r* = -0.699, *P <* 0.001 and *r* = -0.480, *P <* 0.001). These results indicate that hepatic necrosis strongly affected hepatic synthetic function. Other laboratory findings such as initial total cholesterol and platelet levels were statistically correlated with peak PT. The results are due to the coagulopathy and functional damage to liver cells that are characteristic of s-AH. However, HAV is not cytopathic. Therefore, the disease severity may be related to the intensity of the host immune response against HAV.

Low and undetectable HAV RNA load have been reported to be related to FHF and death [9]. Low viremia was suggested to be due to a strong immune response. In contrast, serum HAV RNA titers at admission were higher in patients with s-AH than those with m-AH in our study. In addition, 2 patients with FHF had high viral titers (4.9 and 5.3 log_10_ copies/ml). Unfortunately, only 2 patients with FHF were tested for HAV RNA titer in our study. Sainokami et al. reported no differences in the decline of the viral load between the m-AH and s-AH groups in sequential analyses of viral load [13]. The peak viral load in patients with liver failure may have been underestimated in previous study because they reached the peak viral load before admission [9]. Therefore, our findings do not suggest low viremia is the most important factor associated with liver failure. On the contrary, high viremia may augment a vigorous host immune response, which ultimately contributes to HAV infected hepatocyte injury.

As other previous articles on HAV RNA load have reported, viral load had a significant, negative correlation with PT levels (*r* = -0.453, *P* = 0.001) and a positive correlation with ALT levels (*r* = 0.417, *P* = 0.003) in our study. In addition, peak ALT (HR = 1.001, 95% CI: 1.000–1.001, *P* = 0.004), and HAV RNA titers (HR = 2.076, 95% CI: 1.175–3.669, *P* = 0.012) were independent factors for s-AH in the 48 patients who had HAV RNA titer tested. Based on our results and previous reports, a high HAV RNA titer might be an important viral factor in s-AH [13,21–23].

This study had some limitations. Age was not an independent factor for s-AH in patients who were tested for HAV RNA in contrast to the full study population. This discrepancy might be due to the small number of patients with serum samples stored for HAV RNA titer. The possible relationship between viral load and the mechanism of immune response against HAV has not been defined. Cell mediated immune (CMI) responses, which are associated with HAV infection, may also point to a relationship between viral load and disease severity. Type I IFN could especially influence the strength and effectiveness of T-cell immunity that plays an important role in eliminating HAV infection in humans [24]. Therefore, an HAV-specific IFN assay is needed in the near future to quantify CMI responses according to HAV viral load. Although evaluating viral load at two separate time points is difficult, the results of initial and follow-up HAV RNA titers might yield more information on the interplay between viral load and humoral immunity in patients with acute hepatitis A. If a correlation can be confirmed in a large number of patients, the HAV RNA titer and its dynamic changes would be good markers for predicting s-AH. Finally, although a previous article reported that the HAV genotype and genome variations may not be the main factor in AHA severity, we did not determine HAV genotype or viral sequence variations [25–27].

Although the fatality rate for hepatitis A is generally low, fulminant disease is more common in adults and requires a liver transplantation. Factors that contribute to mortality in patients with s-AH could help physicians prepare for liver transplantation in advance and guide patients to complete recovery without sequelae. If commercial standardized assays for HAV RNA titer were readily available and more feasible in a hospital setting, the prognostic significance of viral load would be well quantified.

In summary, age, peak creatinine, bilirubin, ALT, initial LDH and total cholesterol were independent factors for s-AH in multivariate analysis. In particular, the HAV load was strongly correlated with liver damage in patients with AHA and was an independent factor for s-AH. Our data provide important evidence of a relationship between laboratory findings, including viral load, and clinical outcomes in patients with AHA.
